# Green tea and hyaluronic acid gel enhance fibroblast activation and improves the gingival healing post-third molar extraction

**DOI:** 10.1038/s41598-024-57821-5

**Published:** 2024-03-26

**Authors:** Mariana da Silva Bonatto, Geórgia da Silva Feltran, Thamires Prazeres Barbosa, Davisson Alves Pereira, Samara de Souza Santos, Pedro Gomes Junqueira Mendes, Roberto Sales e Pessoa, Fábio José Barbosa Bezerra, Willian Fernando Zambuzzi, Guilherme José Pimentel Lopes de Oliveira

**Affiliations:** 1 School of Dentistry - Universidade Federal de Uberlândia (UFU), Pará, Av., 1760-1844, Uberlândia, MG 38405-320 Brazil; 2 UNESP - Campus de Botucatu - Instituto de Biociências, São Paulo State University, Rua Prof a. Dr a. Irina Delanova Gemtchujnicov, s/nº, Botucatu, SP 18618-693 Brazil; 3 Centro Universitário Do Triângulo, Uberlândia, MG Brazil

**Keywords:** Tissue regeneration, Bone regeneration, Oral Surgery, Wound healing, Fibroblasts, Green tea, Hyaluronic acid, Randomized controlled trials, Translational research

## Abstract

This study evaluates the effects of a green tea (Camellia sinensis) and hyaluronic acid gel on fibroblast activity and alveolar bone repair following third molar extractions. By examining the gene expression related to cell survival, proliferation, and angiogenesis, the study bridges in vitro findings with clinical outcomes in a split-mouth randomized trial. Human fibroblasts were exposed to the treatment gel, analysing gene expression through RT-qPCR. Twenty participants undergoing bilateral third molar extractions received the test gel on one side and a placebo on the other. Assessments included patient-reported outcomes, professional evaluations, and radiographic analyses at multiple postoperative intervals. The test gel significantly enhanced AKT, CDKs, and VEGF gene expressions, indicating a positive effect on angiogenesis and cell proliferation. Clinically, it resulted in reduced exudate, swelling, and secondary interventions, with radiographs showing improved alveolar bone density after 90 days. The green tea and hyaluronic acid gel significantly improves soft tissue and bone healing post-extraction, offering a promising adjunctive therapy for enhancing postoperative recovery. This gel represents a novel adjuvant treatment option for facilitating improved healing outcomes after third molar extractions, highlighting its potential utility in clinical dental practice.

## Introduction

The surgical extraction of third molars poses significant clinical challenges due to anatomical constraints, such as limited access, varying degrees of impaction, and proximity to critical structures like blood vessels and nerves^1^. These challenges often necessitate complex surgical techniques, leading to postoperative complications including pain, trismus, limited mouth opening, and alveolitis, thereby causing considerable patient discomfort^2^. In response, there has been a growing interest in exploring alternative therapeutic strategies aimed at enhancing tissue regeneration at post-extraction sites^3–5^. Among these, bioactive molecules that can expedite soft tissue healing and bone regeneration are increasingly recognized for their potential to improve the postoperative recovery of patients undergoing third molar extractions^2,6,7^.

Herbal medicines, known for their anti-inflammatory properties, have shown promising results in controlling postoperative inflammation and enhancing healing, with green tea-based compounds standing out for their preclinical efficacy in reducing bone resorption and inflammatory markers in periodontal disease models^8–15^. The antioxidant properties of green tea have also been clinically validated to improve periodontal health more effectively than traditional toothpaste formulations^16^, and systematic reviews have equated the anti-inflammatory efficacy of green tea mouthwashes with that of chlorhexidine^17^. Similarly, green tea's ability to downregulate pro-inflammatory cytokines has been demonstrated in keratinocyte cultures^18^.

Hyaluronic acid, another key molecule in tissue repair, has shown promising results in the management of oral inflammatory conditions and wound healing due to its significant role in connective tissue repair and anti-inflammatory effects^19^. Its application in mouthwashes and gels has been effective in reducing inflammation and preventing complications such as dry socket following dental extractions^20–24^.

Considering these findings, our randomized clinical trial aims to evaluate the effectiveness of a gel formulation combining green tea and hyaluronic acid in promoting mucosal healing after third molar extractions. This study seeks to determine whether such a gel can significantly enhance the healing process, thereby offering a beneficial adjuvant therapy for dental surgeries. We hypothesize that there will be a discernible difference in healing outcomes between sites treated with the green tea and hyaluronic acid gel and those treated with a placebo.

## Material and methods

### In vitro methodologies

#### Cell culture and compounds

Primary human gingival cells were purchased from ATCC (HGF, ATCC PCS-201-018). The cells were grown in Dulbecco's Modified Eagle Medium (DMEM), supplemented with 10% Fetal Bovine Serum (FBS, Vitrocel, Campinas, SP, Brazil) and antibiotics (100 U/mL penicillin, 100 mg/mL streptomycin), in an environment maintained at 37 °C, 5% CO_2_, and 95% humidity. For experimental treatments, cells at a density of 1 × 10^5^ cells/well in 6-well plates were exposed for 24 h to media supplemented with 10% concentrations of hyaluronic acid (Henrifarma, São Paulo, Brazil) and green tea extract (Camellia sinensis) (Chemspecs, São Paulo, Brazil), evaluating their synergistic effects on fibroblast activity without inducing cytotoxicity.

#### mRNA obtaining samples and gene expression

After 24 h of exposition, samples were harvested and mRNA extracted using Ambion TRIzol Reagent (Life Sciences—Fisher Scientific Inc, Waltham, MA, USA) and thereafter treated with DNase I (Invitrogen, Carlsbad, CA, USA). Quantification was performed using a plate reader (SYNERGY-HTX multimode reader, Biotek, USA). Then, cDNA synthesis was carried out using the High-Capacity cDNA Reverse Transcription Kits from Applied Biosystems (2.0 μL 10 × RT Buffer, 0.8 μL 25 × dNTP Mix (100 mM), 2.0 μL 10 × RT Randon Primers, 1.0 μL MultiScribleTM Reverse Transcriptase, 4.2 μL Nuclease- Free H_2_O). The qPCR products were performed on the QuantStudio^®^ 3 real-time PCR equipment (Thermo Fisher Scientific, Waltham, Massachusetts, USA) in a total of 10 μL, containing 2 × PowerUp™ SYBR™ Green Master Mix (5 μL) (Applied Biosystems, Foster City, CA), 0.4 μM of each primer, 50 ng of cDNA and nuclease-free H_2_O. Gene expression was analyzed using control cells by the ΔΔCT method, using β-ACT, GAPDH and 18S13 as housekeeping genes. The ΔΔCT method was used to calculate the values of expressions.

### Clinical strategies

#### Study design

This study consisted of a double-blind, split-mouth, randomized clinical trial study that followed the CONSORT (Consolidated Standards of Reporting Trails) protocol. For statistical purposes, the patients were considered as individual cases and each one of them were submitted to the split-mouth model, in which the same was submitted to the test and control treatment. The sample consisted of 22 (twenty-two) participants from Inpes Odontologia (Uberlândia, Brazil) who underwent extraction procedures for the four third molars and a subsequent procedure to stimulate healing of the alveolar soft tissue after extraction by applying a gel containing green tea and hyaluronic acid over the mucosa of the surgical site. All the patients read and signed an informed consent agreeing to participate in this study. Furthermore, this study was approved by the Research Ethics Committee of the Federal University of Uberlândia with opinion number 48475621.0.0000.5152, and was conducted in accordance with resolution 466 of 12/12/2012 of the National Health Council of the Ministry of Health of Brazil and with the Declaration of Helsinki. The protocol of this study was registered in the REBEC (Brazilian Registry of Clinical Trials) under the number RBR-8z4ctxq (Approval date 12/06/2021—Protocol available at https://ensaiosclinicos.gov.br/rg/RBR-8z4ctxq).

### Sample calculation

The sample calculation was performed based on the study by Heo et al. 2002^25^, which carried out fractal analysis of bone in the regeneration phase after orthognathic surgery. It was found that a fixed mean difference of 0.10 points as being clinically relevant with an expected mean standard deviation of 0.05, a sample size of 20 patients would be sufficient to determine this level of difference with a β power of 0.90 and an α power of 0.05.

#### Inclusion and exclusion criteria

Inclusion criteria comprised individuals over 18 years of age, good oral hygiene (plaque index < 20%) and presence of four third molars with indication for tooth extraction. The tooth extraction was indicated if the 3^rd^ molar was included into de bone and soft tissues, or if teeth are out of position with no occlusion with the antagonist teeth. Exclusion criteria included individuals with periodontal disease, use of drugs that alter bone metabolism, decompensated diabetics (glycated hemoglobin above 8%), heavy smokers (over 10 cigarettes a day), pregnant or lactating women.

#### Surgical procedure and study groups

All participants underwent an initial panoramic clinical and radiographic examination, in order to obtain the necessary data for surgical planning, in addition to subsequent classification of third molars according to Pell & Gregory^26^ and Winter^27^. The surgical procedure was performed under local anesthesia (neural block technique in the area of operation with Articaine 4% associated with epinephrine 1:100,000) and the four third molars were extracted at the same session, using sulcular flaps at the 3rd and 2nd molar associated with release incisions at the buccal-mesial surface of the 2nd molar, and instruments depending on the technical need. After removing the teeth, the flap was sutured with 4–0 nylon thread, and the gel corresponding to each surgical site was deposited on the surgical wound, in sufficient quantity to cover the surgical site (~ 3 ml per socket). The time of the surgical procedure was recorded from the time of application of the anesthetic until the completion of the suture.

**Figure 1:**
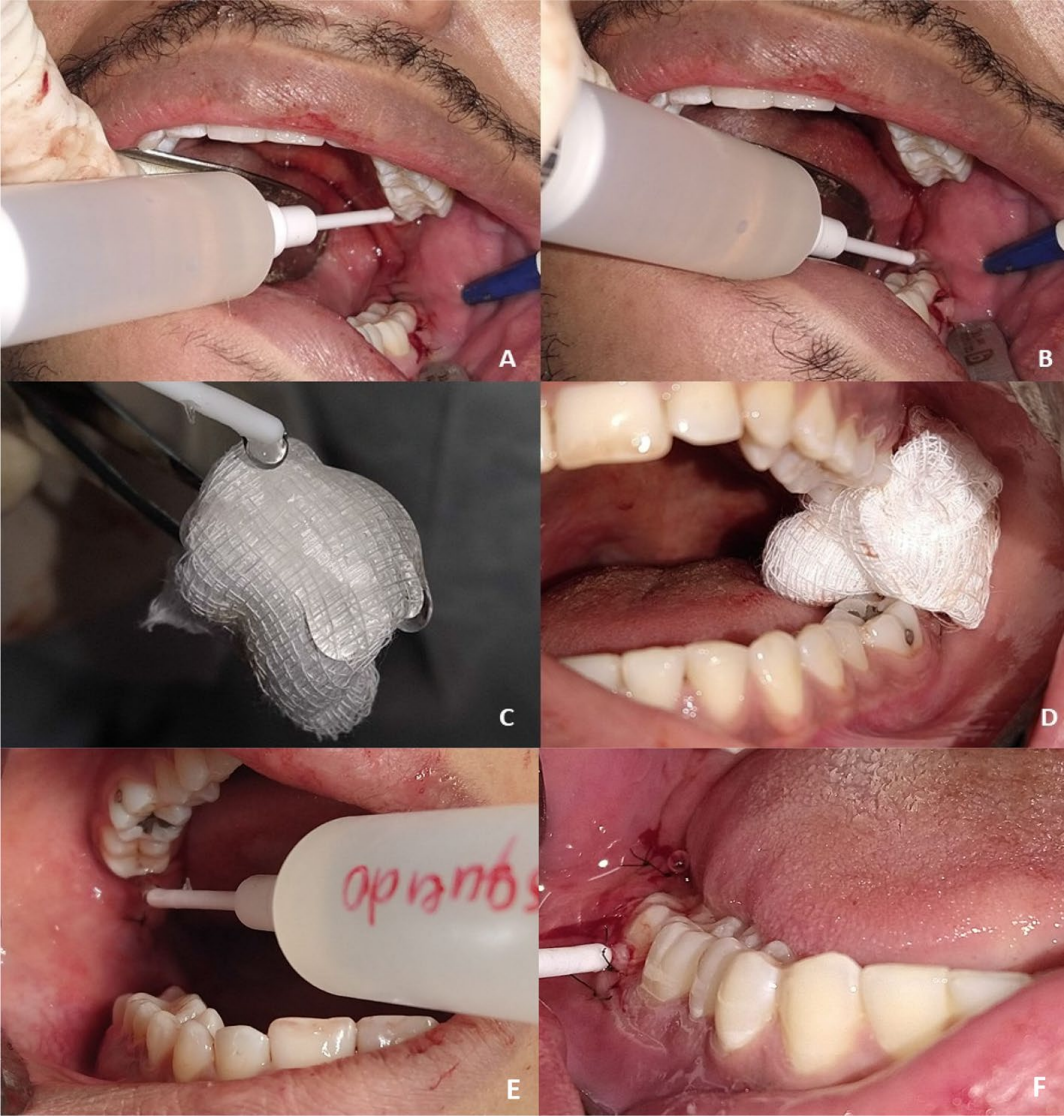
Example of the application of the gel. (**a**) Topic application of the gel on the upper region, (**b**) Topic application of the gel on the lower region. These maneuvers were performed by the patients. (**c**) Just after the surgery, a gauze was soaked with the gel, (**d**) the patients were instructed to bite it, (**e**) during the 3-day period of follow up the patients were reinstructed in the use of the gel, and (**f**) the gel was applied on the entire surgical site.

The selection of treatment in each alveolar mucosa occurred prior to the procedure through an online randomization table (random.org) (GJO). The sides were distributed between the test and control groups according to the type of treatment that they would undergo after the extraction of the 4 third molars, in a split-mouth model. The surgical wound of the maxillary and mandibular molars on the test side was filled with hyaluronic acid gel and green tea, while the surgical wound of the maxillary and mandibular molars on the control side was filled with placebo gel, and the gel was held in position with a gauze (Fig. 1). The coding of the gel was known by only one researcher (GJO), so that the patient, the surgeons, and evaluators were blind to the treatment. The patient was instructed to continue using the gels for 7 days, and they should be used topically on the mucosa of the surgical socket 3 times a day, after oral hygiene (~ 3 ml per socket). At the 3-day period the patients were asked to use the gel in front of the examiners, and adjustments on the gel application were made if it is necessary. The patient was instructed not to consume liquids and solids after 30 min of using the gel. The gel container had only the identification of the side that should be used, so the patient was not aware of the test and control gel. The surgery were performed by two experience surgeons (DAP and MSB).

In all participants, the preoperative medication protocol with dexamethasone (8 mg) was performed in a single dose one hour before the surgical procedure. Postoperatively, diclofenac sodium (50 mg) was prescribed for 3 days every 8 h and dipyrone sodium (500 mg) for 3 days every 6 h, all for oral administration. The sutures were removed after 7 days. The patient and the evaluators did not know where the test and placebo gels were applied.

#### Gel composition and characteristics

The gel used in this study had the following composition (New Dental Care, Ribeirao Preto, Sao Paulo, Brazil): Carboxymethylcellulose 1.00%, Glycerin 5.00%, sorbitol 60.00%, sodium benzoate 0.30%, xylitol 0.50%, laurel glucoside 1.50%, sodium lauryl sulfate 0.50%, hyaluronic acid 0.05%, polyvinylpyrrolidone K 30 0.50%, dimethylsilanediol salicylate (DSBC) 0.50%, thixosil 43B 10.00%, thixosil 73 5.00%, tetrasodium pyrophosphate 0.5%, saccharin 0.05%, green tea extract (*Camellia sinensis*) 0.50%, oil of hydrogenated castor 2.00%, EDTA 0.05%, purified water 11.45%. The test gel was transparent and presented low-viscosity similar the control gel. The control gel had the same composition of the test gel except with no presence of the active compounds.

#### Clinical analysis

Participants were clinically analyzed at 3, 7, 14, 30 and 90 days after tooth extraction. For the analysis of tissue appearance, the examined area was gently dried with a triple syringe and well illuminated by a light from the dental equipment. Clinical parameters were evaluated through visual inspection in scores from 0 to 3, with bleeding (1—absent, 2—palpation-induced, 3—spontaneous), tissue color (1—100% pink gingiva, 2—< 50% of hyperemic and mobile gingiva; 3—> 50% of hyperemic and mobile gingiva), secretion aspect (1—absent, 2—absent with presence of pronounced plaque around the socket, 3—pronounced), and tissue consistency (1—close to normal, pink; 2—flaccid, red; 3—fragile, greenish/gray—3). All data were recorded at the four surgical sites of each patient.

To assess facial edema, two facial measurements were used, the vertical dimension, which consisted of a line from the lateral palpebral commissure to the gonion, and a horizontal dimension, a line from the lower border of the tragus to the labial commissure. These distances were measured on a malleable tape, transferred and quantified using a digital caliper. Mouth opening was measured through the interincisal distance. Regarding the presence of clinical complications, the participants were questioned and eventual complications were noted, and those that could be clinically observed were identified by visual inspection.

The presence of bone spicules, alveolitis and trismus were also noted, as well as the need for alveoli irrigation (pain associated with pronounced exudate secretion) throughout the postoperative period. All these parameters were evaluated by two blinded, trained and calibrated operators (SSS and PGJM).

As for the patient-centered analyses, each participant evaluated through a visual analogue scale (0 to 10, being: 0—none, 1 to 3—little, 4 to 6—fair, 7 to 9—average, 10—excessive) the parameters: pain, inflammation and swelling, hemorrhage and bleeding, difficulty in chewing, and limitation of mouth opening.

#### Radiographic analysis

Panoramic radiographs were used to assess bone tissue structure and density 3 months after tooth extraction. The fractal dimension is a mathematical technique that allows the quantification of complex structures and evaluates the level of irregularities of shapes and objects, its value being directly proportional to its complexity. For this analysis, a region of interest (ROI), in the area corresponding to the extracted tooth, was selected at each of the four sites in the image, thus corresponding to each of the third molars. The box counting method was used to perform the fractal analysis. First, an image of each ROI was taken from the original image. Then, these images underwent the following steps: duplication; applying a Gaussian filter at 35.00 to remove wide-scale variations such as overlaps; subtraction of the second image by the first; adding gray values to 128; image transformation into binary; erodization and dilation for noise removal; and skeletonization, step in which the trabecular bone delineation took place. The fractal dimension values were measured with the software's box-counting function. The image was overlaid with squares of 2, 3, 4, 6, 8, 12, 16 pixels in size. The number of squares that surrounded the trabeculae and the total square count were measured for each pixel of different size. The graph of the logarithmic scale of the values was drawn and the value of the fractal dimension was calculated by measuring the slope of the formed line aligned to the points plotted on the graph, providing a final D-value. To minimize the chances of error in this image treatment process to obtain the final D value, a macro was used that was programmed to execute all the steps, after the ROI cut was performed (PGJM).

### Statistical analysis

The GraphPad Prism 8 software (San Diego, CA, USA) was used to perform the statistical analysis of this study. The results obtained in vitro were plotted as mean ± standard deviation and selected by one-way analysis of variance (ANOVA) with Tukey's post-test, to compare all pairs of groups, where p < 0.05 was considered statistically significant. Clinical outcomes were analyzed as follows: numerical data were evaluated in terms of their distribution using the Shapiro–Wilk normality test. The assessments of the VAS scale and of the healing analyzes were not distributed according to normality and due to this, the Wilcoxon non-parametric test was used to compare the different groups within each evaluation moment. Longitudinal data within each group were statistically compared using Friedman's test complemented by Dunn's test. The data from the analysis of facial measurements were analyzed by means of the parametric paired t-test for comparison between groups and by the Repeated Measurements ANOVA test complemented by Tukey's test for longitudinal evaluation within each group. All statistical tests were applied at a 5% significance level.

### Ethics approval and consent to participate

This study was approved by the Research Ethics Committee of the Federal University of Uberlândia with opinion number 48475621.0.0000.5152,and was conducted in accordance with resolution 466 of 12/12/2012 of the National Health Council of the Ministry of Health of Brazil and with the Declaration of Helsinki. The protocol of this study was registered in the REBEC (Brazilian Registry of Clinical Trials) under the number U1111-1269–7482. All patients read and signed a consent form before the enrolled in this study.

## Results

### Synergistic enhancement of AKT gene expression by green tea and hyaluronic acid

**Figure 2:**
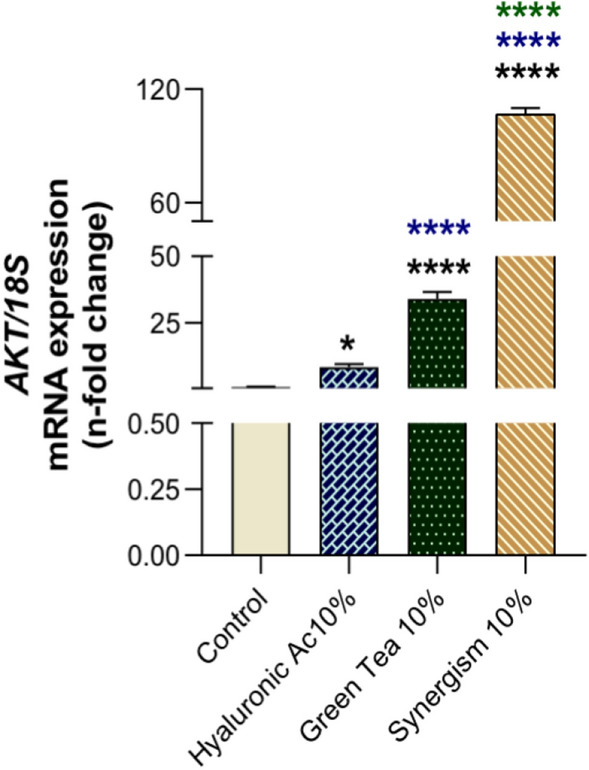
AKT, an important cellular survival biomarker, was evaluated in response to the synergism between hyaluronic acid (10%) and green tea (10%) in human fibroblasts. Control cultures were considered when the cells were maintained under classical conditions, without any treatment. The data shows a significant higher expression of AKT in response to both compounds in synergism. It was considered differences when: *p = 0.0136, and ****p < 0.0001. The value of expression was normalized with 18S data (housekeeping gene).

Our investigation into the combined impact of green tea and hyaluronic acid on fibroblast gene expression revealed a significant modulation of cellular pathways crucial for survival and proliferation. Initial tests confirmed the absence of cytotoxic effects from both compounds, applied independently or in combination, at a 10% concentration (data not shown). Subsequent analyses focused on the expression of the AKT gene, a key indicator of cell survival^28^. The results demonstrated that both green tea and hyaluronic acid individually contributed to the up-regulation of the AKT gene. However, a remarkable synergistic effect was observed when these compounds were combined, leading to an approximately 100-fold increase in AKT expression compared to the control group (Fig. 2). This profound enhancement underscores the therapeutic potential of combining these natural compounds to activate cellular mechanisms that promote tissue healing and regeneration. The statistical significance of these findings is detailed in the accompanying analysis (Fig. 2).

**Figure 3:**
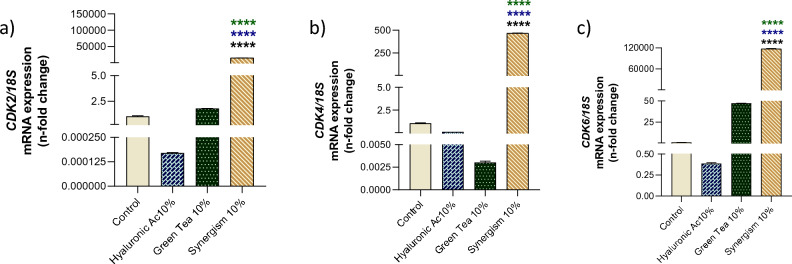
Cell growth related genes were significantly affected by the synergism between hyaluronic acid (10%) and green tea (10%) in fibroblasts. Control cultures were considered when the cells were maintained under classical conditions, without any treatment. The value of expression was normalized with 18S data (housekeeping gene). *CDK* Cyclin Dependent Kinase. ****p < 0.0001.

Further investigation into the synergistic effects of green tea and hyaluronic acid revealed their influence on genes critical for cell cycle progression. Our findings indicate a significant upregulation of key genes associated with cell proliferation, including CDK2 (Fig. 3a), CDK4 (Fig. 3b), and CDK6 (Fig. 3c), when fibroblasts were treated with a combination of these compounds. This up-modulation points to an enhanced capacity for cell proliferation, underlining the therapeutic potential of this synergistic approach for tissue repair and regeneration.

### Enhancement of angiogenesis through VEGF modulation

**Figure 4:**
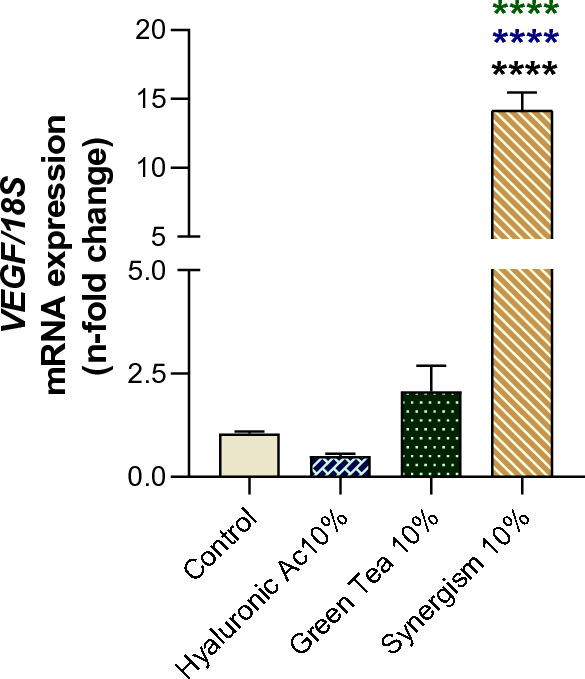
VEGF, an important growth factor to drive angiogenesis, was higher in response to the synergism between hyaluronic acid (10%) and green tea (10%) in human fibroblasts. Control cultures were considered when the cells were maintained under classical conditions, without any treatment. The value of expression was normalized with 18S data (housekeeping gene). *VEGF* vascular endothelial growth factor. ****p < 0.0001.

We have further explored the synergistic interaction between green tea and hyaluronic acid on angiogenesis, a critical process in tissue regeneration^28^. We specifically examined the expression of the Vascular Endothelial Growth Factor (VEGF), known for its pivotal role in angiogenesis^29^. The combined treatment significantly upregulated VEGF expression in fibroblasts, indicating a potent enhancement of the angiogenic potential (Fig. 4). This finding highlights the synergism's effectiveness in promoting angiogenesis, suggesting a promising avenue for improving tissue repair and healing processes through enhanced vascular formation.

### Clinical outcomes

**Figure 5:**
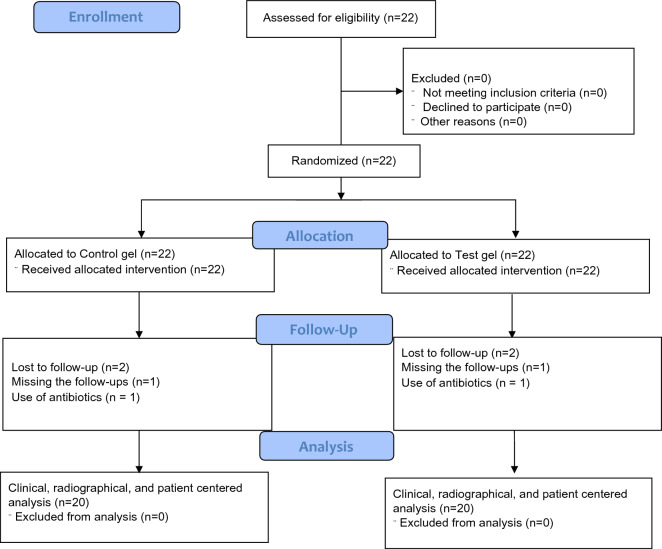
Flow diagram of this study.

The patients were enrolled between Jan/2022 to dec/2022. At the end of the study, 20 patients completed the follow-up and two patients were excluded in this meantime—one of then lost the 3-day follow-up and discontinued the use of the gels, while another patient used antibiotics during the postoperative phase (Fig. 5).

### Green tea gel and hyaluronic acid reduced exudate secretion in post-extraction sockets

A gradual improvement in the clinical aspect of the lesion was verified in both groups with increasing follow-up time, however the test group had a lower alveoli secretion level at 7 days than the control group in lower molars (p < 0.05) (Tables 1 and 2).

**Table 1:** Distribution of scores in the analysis of the clinical aspect of the alveoli after third maxillary molar extraction.

Parameters	Period	Group	1	2	3
Bleeding	3 days	Control	20	0	2
		Test	19	2	1
	7 days	Control	21	0	1
		Test	22	0	0
	15 days	Control	22	0	0
		Test	22	0	0
	30 days	Control	22	0	0
		Test	22	0	0
	90 days	Control	22	0	0
		Test	22	0	0
Secretion aspect	3 days	Control	14	8	0
		Test	18	4	0
	7 days	Control	13	9	0
		Test	15	7	0
	15 days	Control	18	4	0
		Test	20	2	0
	30 days	Control	21	1	0
		Test	20	2	0
	90 days	Control	21	1	0
		Test	22	0	0
Tissue color	3 days	Control	10	12	0
		Test	11	10	1
	7 days	Control	16	6	0
		Test	12	10	0
	15 days	Control	17	5	0
		Test	16	5	1
	30 days	Control	22	0	0
		Test	22	0	0
	90 days	Control	22	0	0
		Test	22	0	0
Tissue consistency	3 days	Control	14	3	5
		Test	14	3	5
	7 days	Control	14	4	4
		Test	18	1	3
	15 days	Control	20	2	0
		Test	19	3	0
	30 days	Control	21	1	0
		Test	22	0	0
	90 days	Control	21	1	0
		Test	22	0	0

**Table 2:** Distribution of scores in the analysis of the clinical aspect of the alveoli after the third lower molar extraction.

Parameters	Period	Group	1	2	3
Bleeding	3 days	Control	18	4	0
		Test	15	7	0
	7 days	Control	19	3	0
		Test	18	4	0
	15 days	Control	20	2	0
		Test	22	0	0
	30 days	Control	22	0	0
		Test	22	0	0
	90 days	Control	22	0	0
		Test	22	0	0
Secretion aspect	3 days	Control	13	9	0
		Test	11	10	1
	7 days	Control	14	1	7
		Test *	20	0	2
	15 days	Control	22	0	0
		Test	21	1	0
	30 days	Control	21	1	0
		Test	22	0	0
	90 days	Control	22	0	0
		Test	20	1	1
Tissue color	3 days	Control	7	15	0
		Test	7	14	1
	7 days	Control	9	12	1
		Test	10	10	2
	15 days	Control	19	3	0
		Test	20	2	0
	30 days	Control	22	0	0
		Test	21	1	0
	90 days	Control	22	0	0
		Test	22	0	0
Tissue consistency	3 days	Control	20	0	2
		Test	19	0	3
	7 days	Control	12	6	4
		Test	17	2	3
	15 days	Control	20	2	0
		Test	20	1	1
	30 days	Control	21	1	0
		Test	21	0	1
	90 days	Control	22	0	0
		Test	22	0	0

*p < 0.05 Lower score values than the control group – Wilcoxon test.

### Patients noted progressive improvement with increasing post-extraction follow-up time, but without differences between treatments.

Regarding the patient-centered analyses, there was a progressive improvement in all parameters tested with increasing post-surgery follow-up time, although there were statistically significant differences between the groups (Table 3).

**Table 3:** Distribution of VAS scale scores applied to the patients.

Parameter	Group	3 days	7 days	15 days	30 days	90 days
Pain	Control	4.36(4.50) ± 2.80	3.63(3.00) ± 2.83	1.09(1.00) ± 1.78	0.25(0.00) ± 0.63	0.09(0.00) ± 0.43
	Test	5.09(5.00) ± 2.38	4.04(3.00) ± 2.59	0.76(0.00) ± 1.17	0.25(0.00) ± 0.55	0.00(0.00) ± 0.00
Inflammation	Control	4.36(4.00) ± 3.09	3.00(3.00) ± 2.61	0.95(0.00) ± 1.96	0.15(0.00) ± 0.36	0.09(0.00) ± 0.43
	Test	4.90(5.50) ± 2.67	2.54(2.50) ± 1.96	0.57(0.00) ± 0.87	0.30(0.00) ± 1.12	0.00(0.00) ± 0.00
Bleeding	Control	3.18(3.00) ± 2.51	1.45(1.00) ± 1.89	0.71(0.00) ± 1.84	0.00(0.00) ± 0.00	0.00(0.00) ± 0.00
	Test	3.13(3.00) ± 2.49	1.45(1.50) ± 1.71	0.19(0.00) ± 0.67	0.00(0.00) ± 0.00	0.00(0.00) ± 0.00
Chewing	Control	5.81(6.00) ± 2.90	3.90(4.00) ± 2.40	1.47(0.00) ± 2.18	0.30(0.00) ± 0.80	0.09(0.00) ± 0.43
	Test	5.77(6.00) ± 2.82	4.04(4.00) ± 2.64	0.90(0.00) ± 1.64	0.15(0.00) ± 0.48	0.00(0.00) ± 0.00
Mouth opening	Control	5.86(7.00) ± 2.29	3.77(4.00) ± 2.81	1.23(0.00) ± 1.99	0.30(0.00) ± 0.92	0.14(0.00) ± 0.65
	Test	5.86(7.00) ± 2.45	3.90(4.00) ± 2.75	0.85(0.00) ± 1.38	0.10(0.00) ± 0.44	0.14(0.00) ± 0.65

### Green tea gel and hyaluronic acid reduced edema up to the 14th postoperative day

A progressive improvement in edema and mouth opening was observed with the increase in the observation period after the extraction surgery. It was also verified that the test group had lower edema values measured by the horizontal dimension of the patients' face up to 14 days-period (p < 0.05) (Table 4).

**Table 4:** Mean and standard deviation data for facial dimensions and mouth opening as a way of evaluating post-extraction edema of third molars.

Parameter	Group	3 days	7 days	15 days	30 days	90 days
Δ Vertical Dimension	Control	0.09 ± 0.83	0.13 ± 1.03	− 0.15 ± 1.04	0.08 ± 0.95	0.05 ± 0.70
	Test	− 0.06 ± 0.87	0.18 ± 0.93	0.06 ± 1.16	− 0.07 ± 0.84	0.03 ± 0.94
Δ Horizontal dimension	Control	0.30 ± 0.81	0.27 ± 0.62	0.09 ± 0.65	− 0.35 ± 0.49	− 0.38 ± 0.64
	Test	0.06 ± 0.73 *	− 0.01 ± 0.59 *	− 0.07 ± 0.64 *	− 0.10 ± 0.54	− 0.30 ± 0.56
Δ Mouth opening reduction	–	17.43 ± 13.10	13.22 ± 13.41	4.91 ± 12.34	− 1.25 ± 6.48	1.65 ± 6.29

*p < 0.05 Lower values than the control group—Paired t-test.

### Post-extraction sockets treated with green tea gel and hyaluronic acid showed higher bone density at 90 days

Radiographic analysis showed that there were no differences between groups and different experimental periods in relation to fractal dimension analysis. However, it was verified that in both groups there was an increase in bone density in the alveoli in the period of 90 days compared to the baseline period, and that the group where the gel based on green tea and hyaluronic acid was applied had higher bone density than the wells treated with the placebo gel within 90 days (p < 0.05) (Table 4).

### Post-extraction socket irrigations were required only in sites treated with placebo gel

No differences were observed between groups regarding the complexity of extracted teeth (Tables 5, 6, 7). There was a need to perform osteotomies (19 in the test gel group and 18 in the control gel group) and sections (14 teeth in each group) in lower teeth with no differences between the groups in relation to these types of surgical maneuvers. The presence of trismus was observed in 5 patients after 3 days of the surgical procedure and in one patient after 7 days of the surgery. Irrigations were performed in one patient in a period of 3 days and in four patients in a period of 7 days in mandibular molar alveoli that was noticed fibrinolytic secretion associated with pain and the presence of biofilm. All these wells were previously treated with the control gel.

**Table 5:** Mean and standard deviation data for bone density and fractal dimension analyzed through radiographic assessment of post-extraction sites.

Parameter	Period	Control		Test	
		Superior	Inferior	Superior	Inferior
Bone density	Baseline	117.5 ± 21.28	113.3 ± 18.60	117.1 ± 21.18	114.0 ± 19.63
	90 days	132.8 ± 16.01^#^	128.9 ± 14.63^#^	130.3 ± 14.47^#^	133.5 ± 12.57*^#^
Fractal dimension	Baseline	1.24 ± 0.10	1.32 ± 0.12	1.21 ± 0.10	1.29 ± 0.11
	90 days	1.26 ± 0.09	1.27 ± 0.09	1.24 ± 0.12	1.27 ± 0.12

*p < 0.05 Higher values than the control group.

^#^Higher values than baseline period – Paired t-test.

**Table 6:** Distribution of the complexity level of teeth extracted in this study according to Winter's classification.

Position/Group	Control		Test	
	Superior	Inferior	Superior	Inferior
Vertical	13	9	14	9
Mesio angulated	3	5	1	5
Disto angulated	4	2	5	2
Horizontal	–	4	–	5
Total	20	20	20	20

**Table 7:** Distribution of the complexity level of teeth extracted in this study according to the Pell & Gregory classification.

Position/Group	Control		Test	
	Superior	Inferior	Superior	Inferior
A	10	9	9	9
B	3	11	5	10
C	7	–	6	1
Total	20	20	20	20

## Discussion

Regenerative mechanism is related with original morphofunctional capacity of cells in renewing the loss/injured tissue. In dentistry this mechanism is widely observed during third molar extraction or implant-related procedures^30–32^. It is known that the wound healing in those cases is critical to prevent the invasion of microorganisms present in the oral microbiome into other tissues and further establishing a chronic infection^33^. Specifically, gingival wound healing comprises the growth of fibroblasts and consequent maturation and remodeling of extracellular matrix (ECM)^31,34–36^. In this way, to identify novel compounds to trigger this mechanism is very welcome for accelerating regenerative events and this study looked for understanding the synergism effect of green tea and hyaluronic acid on regeneration of soft tissue; it seems important to supplement fibroblasts by stimulating them to proliferate during the wound healing^37^. The workplan was based on the analysis of both compound in modulating specific gene expression of human fibroblast and later validate these findings in clinical strategies during third molar extraction related soft tissue healing.

Our experimental study conducted in vitro shows the effect of the compounds on fibroblast phenotype by promoting the overexpression of genes related with cell cycle progression, such as CDKs 2,4, and 6^38,39^. In other words, the synergism of green tea and hyaluronic acid affect the growth of fibroblast and suggest a positive effect in regeneration of soft tissues, such as mucosa present on the oral environment. In fact, evaluating the effect on fibroblasts in vitro is an important starting-point strategy mainly looking for reducing the experimental animal’s number in pre-clinical stages^40^. Moreover, fibroblasts are the main connective tissue cells and responsible to release molecules related with ECM constitution such as collagen fibers, desired during the repair of injures sites^41–43^. Nowadays, based on the well-known function of these cells in remodeling tissue, and more credit has been deposited in fibroblast during pro-regenerative events, mainly by interfering on the regenerative related microenvironment by releasing bioactive molecules such as growth factor or mitogens^44,45^.

**Figure 6:**
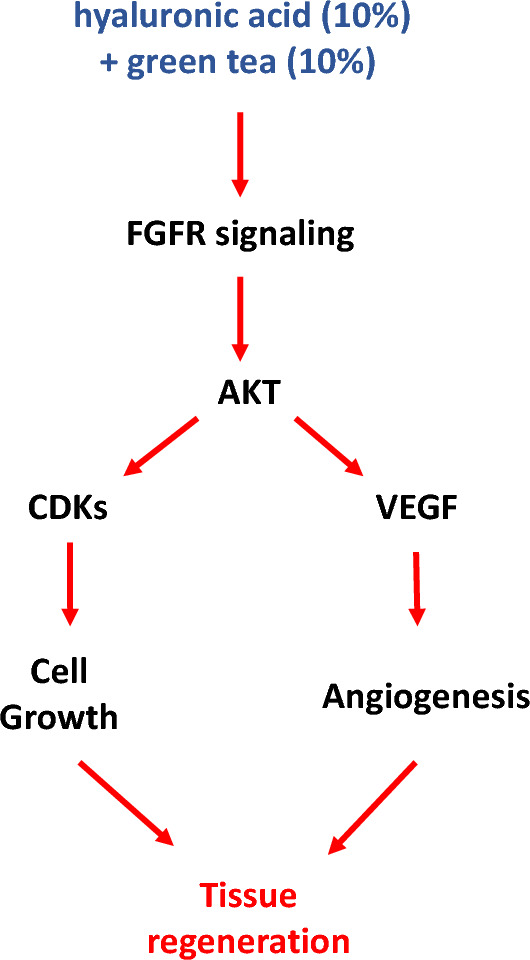
Summary of the main findings found in this study. Note that the conjunction of signaling promoted by the synergism led to the activation of AKT as a hub to drive cellular events related with cell growth, survival, and stimulus for angiogenesis; Taken into account, this signaling pathway is related with connective tissue regeneration, and fibroblast is a pivotal cell in this scenario.

Complimentarily, this study has shown also that challenged fibroblast with green tea and hyaluronic acid significantly over-expresses VEGF (*Vascular Endothelial Growth Factor;* ~ 18 fold-changes). VEGF is one of the most significant factors during the entire angiogenesis^46,47^. From our point of view, challenged fibroblast creates a natural scaffold that is able to drive the regeneration steps, such as the angiogenesis. Angiogenesis is related with the success of regenerative mechanisms guaranteeing nutrients to metabolically active cells in response to increase in concentration of pro-angiogenic factors produced by the fibroblast. A summary of the main findings found in vitro is assembled in the Fig. 6.

Importantly, this study brings a very interesting strategy to investigate whether green tea and hyaluronic acid are stimulators of soft tissue regeneration by associating 2 different approaches: a pre-clinical analysis by evaluating the biology of fibroblasts on this response, and the second looking for validating (or not) these findings in clinical outcomes. Now, considering the clinical outcomes, it is important to mention that the present study demonstrates that a gel-based on green tea and hyaluronic acid modifies the healing course in post-extraction sockets of third molars, reducing the secretion of inflammatory exudate in the lower sockets, less edema, lower frequency of complications and higher bone density. Furthermore, there were no report of side effects regarding the gel use, which showed the safety of the test gel in the treatment of oral surgical sites. Based on these data, the null hypothesis of this study was rejected.

The reduction in inflammatory exudate and in the extraoral edema associated with the use of the test gel can be explained by the previously described antibacterial and anti-inflammatory effects of green tea and hyaluronic acid. Previous studies have indicated that green tea is capable of inducing the secretion of human beta-defensin (hBD) by epithelial cells, protecting these cells from proteolytic degradation and increasing the barrier function of a gingival keratinocyte against the invasion of periodontopathogens^48–50^. Furthermore, green tea was previously shown to reduce the extent and intensity of the inflammatory response, as well as the pro-inflammatory cytokine pattern when used as adjunctive therapy to scaling and root planning^12^. The catechins contained in green tea are responsible for decreasing pro-inflammatory cytokines such as Il-1β and TNFα, in addition to inhibiting peptidase and collagenase activity^8^. Important, this organized microenvirnment promoved by the green tea and hyaluronic acid synergism led to driving the fibroblast performance.

On the other hand, the hyaluronic acid included in the composition of the gel may also have had an influence due to its range of beneficial biological effects. An in vitro anti-plaque effect of hyaluronate (0.025%) similar to that of chlorhexidine was demonstrated in inhibiting bacterial growth^51^. These results are also in agreement with a systematic review that demonstrated benefits in the use of hyaluronic acid in the treatment of periodontal disease, especially due to reductions in plaque index and bleeding on probing^52^, and these effects may be associated with reduced expression of pro-inflammatory cytokines as demonstrated in an in vitro study in fibroblast cultures stimulated by *P.gingivalis* in which hyaluronic acid suppressed the MAPK and NF-κB signaling pathways^53^.

The alveoli that presented pronounced secretion associated with pain were treated by irrigation with saline solution to wash the post-extract sockets and remove food debris, and inflammatory exudate. In fact, the need for this type of management was verified in 5 lower post-extraction wells, all of which had been treated with the control gel. The higher bone density in the group of alveoli treated with the test gel may have occurred due to the absence of mechanical manipulation of the lower alveoli with irrigation. However, hyaluronic acid has also been reported to increase bone formation in areas grafted with osteoconductive bone substitutes in humans^54^, as well as in post-extraction sockets of teeth with periodontal and endodontic damage in dogs^55^. In addition, the catechins present in green tea have also shown an osteoprotective effect by reducing osteoclastogenesis and stimulating differentiation of undifferentiated mesenchymal cells into osteoblasts^56^, and these effects on bone tissue have been indicated to be the reason for the success in the application of green tea in the treatment of periodontitis^16^, and in the prevention of osteoporosis^57^. However, the effect of the association of these two compounds on bone metabolism still needs to be better described.

Other local therapies have been applied to treat lesions in the oral cavity. It has been shown that hyaluronic acid gel enriched with amino acids^58^, chlorhexidine-based gels^59,60^, and the use of blood concentrates^2,61^ positively alter healing in sockets after extraction of third molars^2,58,59^, periodontal bone defects^61^, and soft tissue injuries^58,60^. Thus, the possibility of therapeutic choices to accelerate healing of wounds in the oral cavity is great and the comparison of different therapies is necessary in order to indicate the best therapy for each clinical situation.

The patient centered outcomes of pain, inflammation, bleeding, masticatory function and mouth opening, despite being better on the side that received the test gel, were not statistically different when compared to the control side. The analysis by the patient can often be subjective, making it difficult for the patient to compare certain parameters. This factor could partially contribute to the results found. Some proposed factors could explain these results. Furthermore, analgesic and anti-inflammatory medications prescribed pre and postoperatively directly influence the initial healing phase of the surgical wound, reducing or eliminating pain and mediating inflammation through the reduction of prostaglandins^1^. Thus, since the model of this study was split-mouth, the drug effects would act both on the test side and on the control side, not causing important differences between the sides in the patient's perception. The aforementioned aspects can be considered limitations of this study.

Another important limitation of this study is that the patients self-applied the gel, and this aspect was not controlled. It is worth mentioning that the ability of these patients to apply the gel in a region of difficult access in the oral cavity may have influenced the therapeutic potential of the gel. Furthermore, information regarding gel retention in the oral cavity is still needed in the future. However, the patients were blinded about the type of gel used, and any difficulties encountered in applying the gel must have influenced the results of this study with a similar impact at the control and test sides. Regarding the analysis performed in this study, the radiographic 3D analysis is more accurate method to evaluate the bone formation than the 2D analysis. Anyway, despite the limitations, the bone density and fractal dimension analysis are methods used before to analyze bone healing in panoramic radiographies. In addition, it is important to state that the split-mouth design reduce the effects of the methods errors on the outcomes. Finally, the exclusion criteria in this study impaired the participation of individuals with characteristics with delays the healing of the soft and hard tissues healing. Even soft smokers were not included since no patient with these characteristics were enrolled, despite the intention to include them if they where sorted. Anyway, the effects of the hyaluronic acid and green tea gel on the healing in patients with risk factors (eg. Smokers, diabetics) must be tested in future.

## Conclusion

In conclusion, our study provides compelling evidence that the combination of green tea and hyaluronic acid gel significantly enhances both soft tissue and bone healing post-third molar extraction. The synergistic effect of these compounds led to notable improvements in angiogenesis, cell proliferation, and reduced inflammatory response, as evidenced by gene expression analyses and clinical outcomes. These findings suggest that the green tea and hyaluronic acid gel represents a promising adjunctive therapy for improving postoperative recovery in dental surgery. Future research should focus on further elucidating the molecular mechanisms underlying these beneficial effects and exploring the potential for broader applications in tissue regeneration and wound healing.

## Data Availability

The datasets generated and/or analysed during the current study are available in the Universidade Federal de Uberlândia repository https://repositorio.ufu.br/handle/123456789/35802, however if not accessible data is available from corresponding author on request.
